# Silent microscopy to explore a brain that hears butterflies’ wings

**DOI:** 10.1038/s41377-022-00843-3

**Published:** 2022-05-17

**Authors:** Shin-Ichiro Terada, Masanori Matsuzaki

**Affiliations:** 1 Department of Physiology, Graduate School of Medicine, The University of Tokyo, Tokyo, Japan; 2 Brain Functional Dynamics Collaboration Laboratory, RIKEN Center for Brain Science, Saitama, Japan; 3 International Research Center for Neurointelligence (WPI-IRCN), The University of Tokyo Institutes for Advanced Study, Tokyo, Japan

**Keywords:** Ca2+ imaging, Multiphoton microscopy

## Abstract

A silent two-photon laser-scanning microscopy system, which eliminates mechanical vibrations in the audible range, has enabled the detection of auditory cortical neurons with responses at sound pressure levels as low as 5 dB in nonhuman primates

Since it was first developed^1^, two-photon laser-scanning microscopy (TPLSM) has undergone continuous improvements. TPLSM has been applied to biological research to reveal the spatiotemporal dynamics of cells and molecules within living tissues and/or organisms. In neuroscience studies investigating information processing in neural networks, TPLSM has frequently been used to image genetically encoded calcium indicators (GECIs) to simultaneously detect the activity of numerous neurons at the level of a single neuron or synapse. In 2021, it became possible to measure the activity of approximately 50,000 neurons in a single recording^2,3^. Two-photon calcium imaging has been applied to various species, such as worms, flies, fish, and mice. Recently, studies have begun on its practical applications to nonhuman primates^4–6^, such as common marmosets and macaque monkeys, that share basic brain cytoarchitecture and functions with humans^7,8^.

Vocal communication is well developed in the common marmoset^7^. Recognizing each conspecific voice among the noise of the rainforest is essential for their survival. However, in investigations of how the auditory system recognizes subtle sounds, sounds emanating from the TPLSM can be a major issue. The most widely used scanning device for TPLSM is a combination of a galvo scanner on the slow axis and a resonant scanner on the fast axis. Typical TPLSM has a resonant-scanner speed of 8 kHz (approximately 30 frames per second [fps]) and a wide scanning angle of 26°. However, because of its resonant nature, TPLSM produces loud single-frequency acoustic noise at 8 kHz, which falls within the audible range of many animal species. This contrasts with the visual system, where TPLSM uses a 900–1100 nm infrared laser that is outside of the visible range, thus creating a completely dark condition for animals. Because the gray matter of monkeys has 60% higher absorbance than that of rats^9^, the availability of high laser power is important for imaging the nonhuman primate cerebral cortex. Moreover, imaging awake animals inevitably causes motion artifacts due to body movements, and generally, two-dimensional (2D) full-frame raster scanning is preferred for offline motion correction. Therefore, auditory studies in nonhuman primates require reducing the noise induced by microscopy, maintaining high laser power, and performing 2D raster scanning.

Song et al^10^. recently solved these problems by using two acoustic optic deflectors (AODs) as a nonmechanical scanning device (Fig. 1a). An AOD can change the angle of the diffracted beam depending on the frequency of the ultrasound applied to the crystal, and combining two AODs allows for fast 2D scanning of the focal point. However, AODs generate spatial and temporal dispersions of ultrashort laser pulses. Therefore, the authors compensated for these dispersions by using a simple dispersion-compensating optical system with a diffraction grating. Although AODs enable fast random access to multiple targets (i.e., neurons or synapses), it takes approximately 10 μs to reach a steady state every time the frequency is changed. Therefore, if the frequency is discontinuously changed every pixel, it takes >2 s to create one frame of 512 × 512 pixels. Instead, chirping the frequency applied to the AOD at a constant rate is widely used for high-speed raster scanning. However, when chirping the AOD, changes in divergence occur only in the direction of the crystal vibration, and the AOD functions similar to a cylindrical lens, which results in image quality degradation due to astigmatism (Fig. 1b). One method to correct this is to install an additional pair of AODs and chirp in opposite directions to counteract the effect of a cylindrical lens^11^. In addition, two or more AODs can be added to rapidly shift the focal position to achieve wide three-dimensional (3D) scan volumes^12,13^. However, because the diffraction efficiency of AODs is typically around 70% at wavelengths of approximately 940 nm for excitation of GECIs, such as GCaMP, laser power attenuates by a multiple of the number of AODs. In contrast, the reflection efficiency of galvo-resonant scan optics is at least 95%. Song et al. set the same chirping speeds for the two AODs (Fig. 1c), rather than adding other AODs, which enabled matching of the divergence occurring along both axes. Astigmatism was corrected using a divergence compensation lens that was mounted immediately after the AOD (Fig. 1a, b). Despite the simplicity of this design, the depth of the focal point can be changed by adjusting the chirp speed. Thus, although the movable distance was short, 3D scanning was possible (Fig. 1c). The resulting scan optics, with AODs combined with sound insulation for other major noise sources (i.e., laser power supply, chiller, and laser oscillator), enabled the acoustic noise to be kept lower than the perceivable range throughout the audible range of the marmoset.

**Fig. 1:**
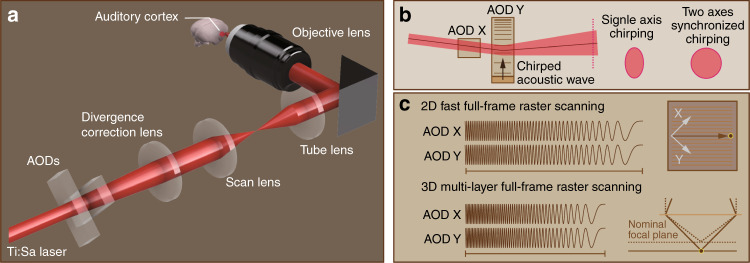
Configuration for silent raster scanning using two AODs. **a** Excitation light from a Ti:Sa laser is transmitted to an orthogonal pair of AODs. The diffraction generated by the AODs with linear chirping of the control frequency enables rapid control of the output angle without generating an audible sound. **b** If only one AOD is chirped, the divergence of the emitted light from the AOD changes only with respect to the chirped axis; however, by chirping the two AODs at the same speed, equal divergence is achieved. The equally changed divergence is corrected by the divergence collection lens and subsequently transmitted to the illumination optics of an ordinal two-photon laser-scanning microscope. **c** A single pair of AODs chirping at the same speed enables raster scanning by scanning the laser in a diagonal direction. By changing the chirping speed, the light coming into the objective lens converges or diverges, and the focal point can be shifted above or below the normal position

In this optical system with a 10 × objective lens, the full frame rate was 23.5 fps, and the field of view comprised 653 × 652 pixels, with a pixel length of 0.585 μm. The lateral and axial full widths of half maximum were approximately 1.5 and 7.8 µm, respectively. These values were equivalent to those obtained using conventional TPLSM with galvo-resonant scanners that was used for imaging the sensorimotor cortex of the common marmoset^4^.

Subsequently, the authors used a silent TPLSM system to perform calcium imaging of the auditory cortex in awake head-fixed marmosets. The activity of each layer 2/3 neuron was measured across a wide range of frequencies (220–7040 Hz) and sound pressure levels (SPLs; 5–65 dB). Following post hoc motion correction, auditory responses were clearly detected in many neurons, which began responding to sounds as low as 20 dB SPL, corresponding to the sound of leaves touching. Although studies using electrical recordings have already demonstrated that each auditory cortical neuron shows a preference for a specific frequency, the authors identified three types of neurons in the auditory cortex: one type that responds more widely as the SPL increases, another type that responds consistently regardless of the SPL, and one type that responds more strongly to specific SPLs. Notably, a subset of neurons showed a maximal response at an SPL as low as 20 dB, rather than at higher SPLs. Moreover, several neurons responded to sounds as low as 5 dB SPL, which is lower than the sound of butterflies’ wings (10 dB). Therefore, silent TPLSM is a promising approach for discovering new phenomena in the auditory cortical processing system.

What are the potential further improvements of silent TPLSM? The main cortical layer that receives sensory inputs from the thalamus (layer 4) is located approximately 400 µm from the surface of the brain in mice, but approximately 1.2 mm in the marmoset. In mice, calcium imaging of the deepest layer (>700 µm deep from the cortical surface) was demonstrated by combining a red GECI and a long wavelength laser of 1100 nm^14^. Similar deep imaging is also possible using three-photon excitation of a red GECI and a 1700 nm laser^15^. In the future, it will be necessary to select appropriate AOD crystals for such wavelengths to increase the depth of imaging in the cerebral cortex in the nonhuman primate. An experimental paradigm to examine the cocktail-party effect, which is related to our ability to extract a specific voice or word from a multitalker scene, in the marmoset has been recently developed^16^. In addition, silent TPLSM is valuable for investigations of the rodent auditory system. Silent TPLSM and future improvements will undoubtedly shed further light on auditory information processing.
